# Contribution of remote *M*.*tuberculosis* infection to tuberculosis disease: A 30-year population study

**DOI:** 10.1371/journal.pone.0278136

**Published:** 2023-01-27

**Authors:** Judith R. Glynn, Palwasha Khan, Themba Mzembe, Lifted Sichali, Paul E. M. Fine, Amelia C. Crampin, Rein M. G. J. Houben

**Affiliations:** 1 Department of infectious Disease Epidemiology, London School of Hygiene and Tropical Medicine, London, United Kingdom; 2 Interactive Research & Development, Pakistan; 3 Malawi Epidemiology and Intervention Research Unit, Lilongwe, Malawi; 4 African Institute for Development Policy, Lilongwe, Malawi; 5 Institute of Health and Wellbeing, University of Glasgow, Glasgow, United Kingdom; 6 TB modelling group, TB Centre, LSHTM, United Kingdom; The 8th Medical Center of PLA General Hospital, CHINA

## Abstract

**Background:**

The importance of remote infection with *M*.*tuberculosis* as a cause of tuberculosis disease (TB) is unclear, with limited evidence of impact on TB rates beyond 10 years. Our objective was to assess rates of tuberculosis over 30 years by *M*.*tuberculosis* infection status at baseline in Karonga District, Northern Malawi.

**Materials and methods:**

Population-based surveys of tuberculin skin testing (TST) from the 1980s were linked with follow-up and TB surveillance in Karonga district. We compared rates of microbiologically-confirmed TB by baseline TST induration <5mm (no evidence of *M*.*tuberculosis* infection) and those with baseline TST >17mm (evidence of *M*.*tuberculosis* infection), using hazard ratios by time since baseline and attributable risk percent. The attributable risk percent was calculated to estimate the proportion of TB in those infected that can be attributed to that prior infection. We analysed whole genome sequences of *M*.*tuberculosis* strains to identify recent transmission.

**Results:**

Over 412,959 person-years, 208 incident TB episodes were recorded. Compared to the small induration group, rates of TB were much higher in the first two years in the large induration group, and remained higher to 20 years: age, sex and area-adjusted hazard ratios (HR) 2–9 years post-TST 4.27 (95%CI 2.56–7.11); 10–19 years after TST 2.15 (1.10–4.21); ≥20 years post-TST 1.88 (0.76–4.65). The attributable risk percent of remote infection was 76.6% (60.9–85.9) 2–9 years post-TST, and 53.5% (9.1–76.2) 10–19 years post-TST. Individuals with large TST indurations had higher rates of unique-strain TB (HR adjusted for age, sex and area = HR 6.56 (95% CI 1.96–22.99)), suggesting disease following remote infection, but not of linked-strain TB (recent transmission).

**Conclusions:**

*M*.*tuberculosis* infection can increase the risk of TB far beyond 10 years, accounting for a substantial proportion of TB occurring among those remotely infected.

## Introduction

Understanding the relative contribution of recent and remote *M*.*tuberculosis* (Mtb) infection to the incidence of tuberculosis disease (TB) is fundamental for control strategies. It is known that TB can occur decades after infection [1], and in areas where TB incidence has fallen, the higher incidence of TB in the elderly, especially that caused by unique strains, can be explained by remote infections acquired in earlier life [2].

Considerable uncertainty remains, however, about the importance of remote infection (currently defined as at least 2 years since exposure) on the burden of disease in areas where infection rates are high. A recent review found few studies with follow-up beyond 10 years, in modern high-incidence settings, or with a comparison between groups infected and uninfected at baseline [3]. The paradigm of lifelong infection has been challenged, and as a consequence, TB policy is shifting to focus on the high risk of disease within the first two years after infection [3–6]. Given the implications for global TB policy and research, further evidence on the long-term impact of Mtb infection, or lack thereof, on the risk TB is crucial.

One reason for the limited evidence is the challenge of developing the required cohorts, reliably recording Mtb infection status at entry, and linking this to both TB and vital status outcomes across decades. Another limitation is the reliance on tuberculin skin tests (TST) or interferon-γ release assays as measures of infection. The far from perfect specificity, especially with the low cut-offs often used [3], will misclassify infection status and underestimate any association between TB and remote infection. In addition, molecular epidemiological data, which can distinguish unique strains of Mtb, which are likely to have arisen from remote infection, from strains shared by other people with TB in the community, which are likely to represent recent transmission, are often unavailable [7].

Using strict cut-offs, we have analysed data from population-based tuberculin surveys and linked studies, including molecular epidemiological data, in Karonga District, northern Malawi to compare the incidence of TB in those with large and small initial tuberculin reactions over three decades.

## Methods

### Baseline *M*.*tuberculosis* infection

Total population surveys were carried out between 1980–4 and 1985–1989 as part of long-term studies of leprosy. The second survey was the recruitment phase of a trial of repeat BCG and killed *M leprae* [8–11]. In each survey a subset of the population selected by area of residence received a tuberculin skin test. These were done before the trial vaccines were given. The tuberculin tests used 2IU of RT23 placed on the volar surface of the forearm, and were read by trained field teams 48–72 hours later. The diameters were recorded along and across the arm and the average induration taken. Individuals found to have large indurations were screened for TB; at that time, as elsewhere, there was no policy of preventive therapy [12].

### TB follow-up

Since the early 1980s, all individuals diagnosed with TB in the district have been recorded as part of epidemiological studies of TB and the vaccine trial follow-up. Case ascertainment relied largely on enhanced passive ascertainment, with field staff based at the hospital and peripheral health centres to screen individuals with chronic cough or otherwise suspected to have TB. Individuals seen in other studies were also asked about cough of more than 3 weeks’ duration. Specimens were processed and smears read in the project laboratory, with cultures sent to the UK for species confirmation [13, 14].

### Vital status follow-up

After the second survey in the 1980s there have been no further total population surveys, but follow-up information is available from other studies conducted in the district, including small population based surveys and demographic surveillance in different areas of the district [15, 16]. All studies use common identifiers for individuals, so the date individuals were seen, or the date they were reported to have left the district or died, as recorded in other studies, can be used to estimate the date individuals were last known to be alive and in the district.

In 2009–2013, individuals recorded as having TST induration sizes of greater than 20mm in the 1980’s surveys were identified. Those who were not already known from continuing studies in the area to have developed TB or to have died or left the district were sought. Interviewers asked those found living in Karonga district about prior treatment for TB and current cough. For those who had left the district or died, a suitable informant was asked about prior treatment for TB as well as dates of departure or death.

No comparable period of intensive follow-up was available for individuals with smaller initial TST induration sizes but we used the data from the other studies in the district [16]. To avoid bias in the follow-up data towards those who developed TB, information on the date when individuals were last known to be alive and in the district was only used from studies that were not related to TB. Only TB that occurred before an individual was recorded in the database for a non-TB related reason was included.

### Definitions

To define groups of individuals who were very likely to have true infections with Mtb and who were truly not infected, we restricted the analysis to those with very large (>17mm) or very small (<5mm) tuberculin reactions, defined from the first TST done in the whole population surveys in the 1980s. While 17mm is a higher cut-off than usually used, we wanted to maximise the likelihood that a positive test reflected a true infection, and non-specific reactions are not thought to get this large [12].

For this analysis TB was considered confirmed if smear or culture or GeneXpert or histology positive, excluding only those with a single scanty smear. Those with only clinical diagnoses (symptoms and/or X-ray) or a single scanty sputum smear were considered to have unconfirmed TB.

HIV status was not known for most of the population, but was known for some of those who developed TB, through testing offered at the time of diagnosis or in other studies [17–19]. Individuals were defined as having HIV negative TB if they had a negative HIV test after the TB episode or up to two years before, with no positive test or self-report in the period up to 6 months after the first recorded contact (diagnostic test or treatment initiation) of the TB episode. Individuals were defined as having HIV positive TB if they had a positive HIV test or self-report at any time before or up to 6 months after the first recorded contact for that TB episode, without an HIV negative test after the start of the TB.

### Statistical methods

We used survival analysis, and present the results as rates and hazard ratios, using Poisson regression. Individuals were considered “at risk” from the time of the skin test. They were censored at the date of first evidence of TB (confirmed or unconfirmed), or the date they were last known alive in the district, or the end of 2016 (when TB surveillance by the project ended), whichever was earliest. Individuals who were known to have been given isoniazid prophylaxis were censored at the date on which it was started.

The aim of the analysis was to assess the magnitude and duration of the increased risk of TB in those with known infection. We therefore investigated the time periods < 2 years, 2–9 years, 10–19 years and ≥20 years after the skin test. Analyses were adjusted for age, sex and area of residence at the time of the skin test. We also explored BCG scar at baseline and BCG vaccination in the trial as possible confounders. We were only able to measure risk by time since the initial skin test, not time since infection. We also investigated age at skin test and sex as possible effect modifiers.

To estimate the proportion of TB in those infected that can be attributed to that prior infection we calculated the attributable risk percent. This is defined as (risk in exposed—risk in unexposed)/risk in exposed. Since this is equivalent to 1 –(risk in unexposed/risk in exposed) it can be calculated from the hazard ratios. For this analysis, ‘risk’ was defined as risk of TB disease, and the exposure was large initial TST.

### Molecular epidemiology

An increased rate of TB among those with remote infections (i.e. those with large initial TST) could reflect a higher risk of recent re-infection with circulating strains, rather than TB from their remote infection. Whole genome sequencing is available on some *M*.*tuberculosis* specimens from Karonga District from September 1995 to 2014. The methods have been described in detail elsewhere [20]. A matrix was drawn up of the number of single nucleotide polymorphisms (snps) between all pairwise combinations of specimens. For this analysis, specimens were classified as “clustered” if there was at least one other specimen in the dataset with ≤10 snps difference. Specimens were classified as *unique* (and therefore likely to be due to reactivation) if they were >10 snps different from any other specimen in the dataset, or if they were the first in a cluster of specimens with up to 10 snps difference. Specimens were classified as *linked* (and therefore likely to be due to more recent transmission) if they were clustered but not the first in a cluster. For the analysis we have excluded instances where the first two specimens in a cluster were collected within one month of each other, as it is difficult to know who infected whom. Since the infecting source will have been missed for many of the people with TB in the first years of the dataset, specimens in the first 3 years of data (before 1999) cannot be classified as unique or linked and have been excluded from the analysis. We estimated rates of unique and of linked TB by baseline TST induration, from 1999, 10 or more years after the skin test.

### Sensitivity analyses

Because of the different follow-up procedures for those with indurations of >20mm and those with smaller indurations, we also analysed cohorts with baseline indurations of 18-20mm and >20mm separately, as a sensitivity analysis.

## Results

### Cohort

Using the rules above we identified 52,633 individuals with initial indurations of <5mm, and 4,634 >17mm (3,005 at 18-20mm and 1,629 >20mm). Follow-up information after the date of the skin-test was available for 43,056 (81.8%) of those with indurations <5mm, and 4,057 (87.6%) of those with indurations of >17mm. The source of the follow-up information was mostly from the population surveys in the 1980s and early 1990s, with further follow-up at the time of the baseline census for the demographic surveillance programme, and subsequent surveillance activities. For those with indurations >20mm most information came from the special follow-up survey (Table 1 and Table 1 in S1 Appendix). Those in the small induration group were much younger: at the time of the TST test two thirds were less than 15 years old, compared to less than 20% of the other groups.

**Table 1:** Individuals included in the different TST induration groups with characteristics and source of follow-up information.

		TST induration			
		<5mm		>17mm	
		N	%	N	%
Identified		52,633	100	4,634	100
Age at initial TST					
	< 15 years	35,443	67.3	765	16.5
	15–29 years	8,867	16.9	1,068	23.1
	30–44 years	4,132	7.9	1,097	23.7
	45–59 years	2,690	5.1	1,175	25.4
	≥ 60 years	1,501	2.9	529	11.4
Sex	Female	29,194	55.5	2,519	54.4
Date of TST					
	1980–84 (1^st^ survey)	36,643	69.6	3,145	67.9
	1985–89 (2^nd^ survey)	15,990	30.4	1,489	32.1
Area or residence (south to north)					
	1 (rural + truck-stop)	6,999	13.3	477	10.3
	2 (rural)	10,434	19.8	780	16.8
	3 (peri-urban)	9,552	18.2	812	17.5
	4 (urban)	8,440	16.0	734	15.8
	5 (rural + trading area)	9,861	18.7	1271	27.4
	6 (rural + border)	7,347	14.0	560	12.1
Follow-up information available		43,056	81.8	4,057	87.6
Last information (for those with follow-up):					
	Whole population survey	28,076	65.2	1,815	44.7
	Sample population surveys	6,249	14.5	429	10.6
	Baseline census	2,896	6.7	198	4.9
	Demographic surveillance	4,079	9.5	211	5.2
	Follow-up of >20mm	0	0	1,253	30.9
	Other	1,756	4.1	151	3.7

### Tuberculosis disease rates

**Fig 1:**
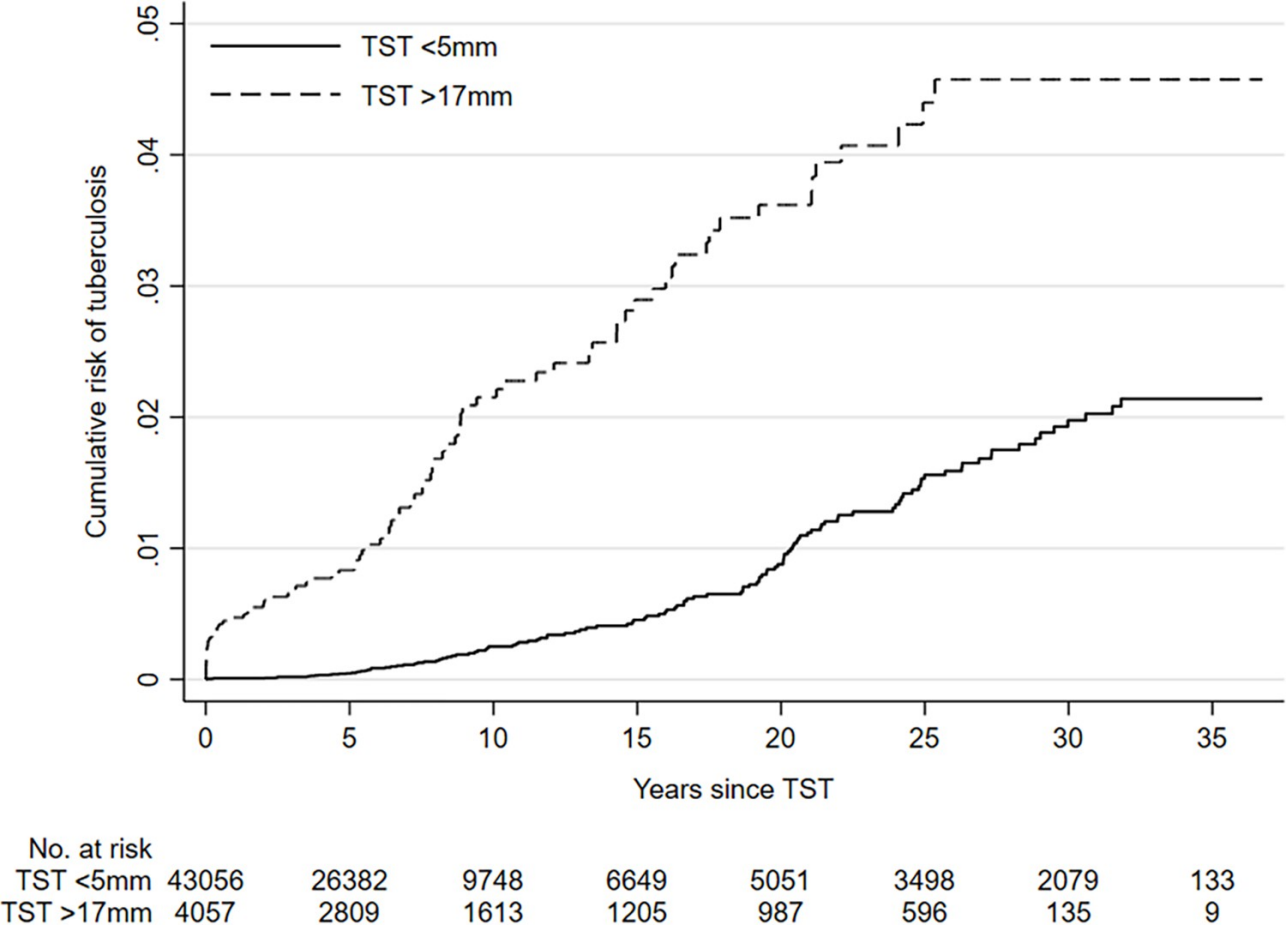
Cumulative risk of tuberculosis by time since tuberculin skin test, and induration size.

The cumulative risk of TB over time is shown in Fig 1, and the rates in the different time periods in Table 2. The large induration group had a very high rate in the first two years. The rate then dropped but remained higher than that in the small induration group until 20 years after the TST.

**Table 2:** Incidence rates of tuberculosis by induration size and time since the tuberculin skin test.

	TST <5mm			TST >17mm		
Time since TST	TB	pyar	Rate/1000pyar (95% CI)	TB	pyar	Rate/1000pyar (95% CI)
< 2 years	4	82,582	0.048 (0.018–0.13)	22	7,826	2.81 (1.85–4.27)
2–9 years	40	174,592	0.23 (0.17–0.31)	36	20,177	1.78 (1.29–2.47)
10–19 years	40	68,690	0.58 (0.43–0.79)	18	12,402	1.45 (0.91–2.30)
≥ 20 years	43	40,769	1.05 (0.78–1.42)	7	5,921	1.18 (0.56–2.48)

pyar = person years at risk, CI = confidence interval

Further analyses compared the rates in the large and small induration groups, adjusted for age, sex, and area (Table 3). Adjusting for age reduced the hazard ratios in the earlier periods, whereas adjusting for sex and area made little difference to the results. Adjusting for BCG scar at baseline and BCG vaccination in the trial made almost no difference to the results so they have not been included in the models to avoid over-parameterisation. After adjustment for age, sex, and area, large indurations (>17mm) were associated with a 4-fold increase in incidence of TB 2–9 years after the skin test, and with a 2-fold increase 10–19 years after the skin test (Table 3).

**Table 3:** Hazard ratios for tuberculosis by time since the tuberculin skin test for those with indurations >17mm compared to those with indurations of <5mm.

Time since TST	HR (95%CI)		
	Crude	Adjusted for age*	Adjusted age, sex and area
< 2 years	58.04 (20.00–168.42)	41.31 (12.72–134.11)	41.85 (12.78–137.04)
2–9 years	7.79 (4.96–12.21)	4.37 (2.65–7.22)	4.27 (2.56–7.11)
10–19 years	2.49 (1.43–4.35)	2.23 (1.19–4.17)	2.15 (1.10–4.21)
≥ 20 years	1.12 (0.50–2.49)	1.37 (0.58–3.22)	1.88 (0.76–4.65)

HR = hazard ratio, CI = confidence interval *Age groups <15 15–29, 30+ years. Adjusting for age in 5 groups (<15 15–29, 30–44, 45–59, 60+ years) gave almost identical results

When analysed separately, results for the two large induration groups were similar. The source of follow-up information for the 18-20mm induration group was comparable to that in the <5mm group (see Tables 1–3 and Fig 1 in S1 Appendix).

**Fig 2:**
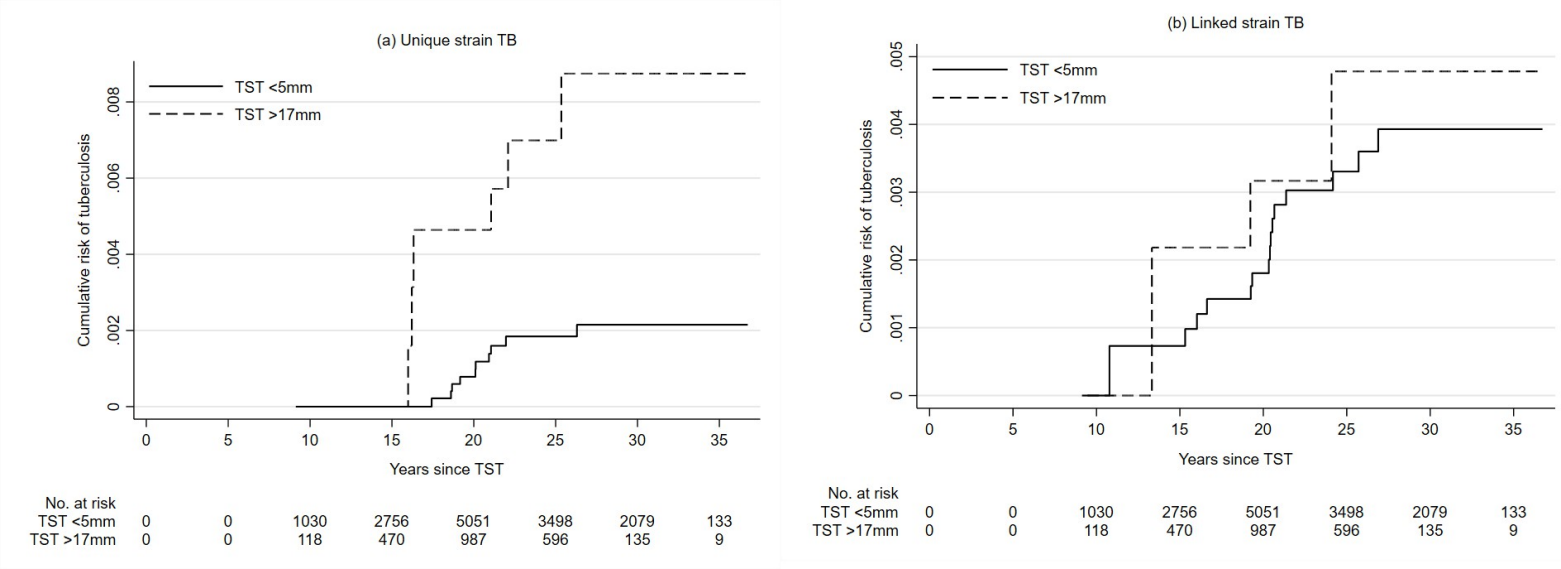
a: Cumulative risk of unique strain tuberculosis after 1998, by time since tuberculin skin test and induration size. b: Cumulative risk of linked strain tuberculosis after 1998, by time since tuberculin skin test and induration size.

HIV status at the time of TB diagnosis was only available for some individuals in the analysis: 73 who were HIV negative at the time of diagnosis and 46 who were HIV positive. After adjustment for age, sex and area, more than 2 years after the TST, compared to those with indurations <5mm, the adjusted hazard ratio for those with indurations >17mm was 2.47 (1.39–4.37) for HIV negative TB, and 2.60 (1.18–6.04) for HIV positive TB. Further detail by time periods is shown in Fig 2 and Table 4 in S1 Appendix.

There was some variation in the results by age at the time of the initial skin test (Fig 3 and Table 5 in S1 Appendix, likelihood-ratio test for interaction of age and TST groups *p* = 0.20). For each time period since the skin test, the association between TST induration and risk of TB was stronger in the younger age groups, though confidence intervals are wide. The association between TST induration and risk of TB was also stronger in females than in males (Table 6 and Fig 4 in S1 Appendix, likelihood-ratio test for interaction of sex and TST groups *p* = 0.38).

### Proportion of TB attributable to remote infection among those infected

The attributable risk percent was calculated using the age, sex and area adjusted hazard ratios in Table 3. This gives an attributable risk of TB among those with remote infection of 76.6% (95%CI 60.9–85.9) for the period 2–9 years after the TST, and 53.5% (95%CI 9.1–76.2) for the period 10–19 years after the TST.

### Molecular epidemiological data

From 1999, 81 people with baseline large or small TST reactions developed TB, of which 16 could be classified as unique and 18 as linked (unknown for 47). The rate of linked strain TB was similar in people who had small or large TST indurations in the 1980s, but the rate of unique strain TB was much higher in those who had large TST indurations (Fig 2). This persisted after adjusting for age, sex and area (HR 6.56 (95% CI 1.96–22.99)), see Tables 7 and 8 in S1 Appendix.

## Discussion

In this large population-based cohort study with decades long follow-up in a high burden area during the chemotherapy era, we show a clear increase in risk of TB in individuals with remote Mtb infection, which persists up to at least 20 years after the TST test. The true duration of persistence of elevated TB risk from remote infections is likely to be even longer, as most individuals will have been infected some years before the initial test.

The estimates of attributable risk of TB in those with remote infection imply that in this population, assuming causality, 77% of the TB that develops 2–9 years after an infection is due to that remote infection and 55% of the TB that develops 10–19 years after an infection is due to that remote infection. These data are supported by our molecular data: the rate of unique strain TB but not linked strain TB was higher in those who had large TST reactions, i.e. the risk of TB from a remote Mtb infection was much higher in those with a large TST induration, and TB due to recent transmission was similar in the two groups.

Many studies define a “positive” TST using a low cut-off such as 10mm, so misclassification is likely [21]. Here we minimised misclassification by only considering those with very small or very large tuberculin reactions. Although some individuals with very large indurations were found on investigation to have previously undiagnosed TB disease (contributing to the high initial TB rates) most were not, suggesting that the large induration indicated a prior Mtb infection not TB disease. Our definitions exclude individuals with TST indurations of 5-17mm. While necessary to reduce misclassification, it is possible that a large TST reflects a biological difference in the body’s response to Mtb infection, which carries through to their risk of disease from a remote infection [22].

While the unknown dates of infection are likely to lead to an underestimate of the true duration of the risk of TB following remote infection, it has been argued that, because reversion of TST positivity occurs, those detected as positive might over-represent those with persistent infection and so overestimate reactivation rates in all who have been infected [3]. This is unlikely to be an important bias as the recent systematic review found lower rates of TB in studies with unknown timing of Mtb exposure than in studies with known timing [3]. Furthermore, while some apparent reversions are likely to be due to test instability and misclassification [12], this is unlikely with the very large induration size cut-off used in our study. As part of the 2009–2013 follow-up of individuals with TST > 20mm in the surveys done in the 1980s, 101 HIV negative participants had repeat skin-tests. All but 4 (96%) had indurations ≥10mm, and 25 (25%) still had indurations > 20mm (unpublished results).

Absolute rates of TB will be underestimated in our study due to the passive ascertainment of TB and the use of strict diagnostic criteria, however the hazard ratios should not be biased. We assessed adjustment for age, sex, area, and BCG status, but could not make adjustments for other possible confounders, such as socioeconomic status, as these were not measured over the period of the study. It is possible that individuals in higher risk settings (e.g. social contact networks) that accounted for their initial infection were more likely to continue to live in higher risk settings and therefore have a higher risk of reinfection [23, 24]. However the molecular data strongly suggest that the persistent increased risk of TB in those with large prior indurations more than 10 years previously is not due to them being at continued increased risk of re-infection but to persistence of their original infection. Although whole genome sequencing data was only available for some of the follow-up period, limiting the power of the analysis, and for a minority of people with TB, so some misclassification of linkage is likely [25], this should not be biased by baseline TST induration. The molecular results support the main analysis, and provide further evidence that the risk of reactivation of Mtb infection persists well beyond 10 years.

HIV was not known for most of the population, but restricting the outcome to HIV negative TB also showed a clearly higher risk of TB beyond 2 years of follow-up in those with large indurations. (The low rate of HIV positive TB initially is an artefact due to the lack of testing in the 1980s as well as low HIV prevalence at that time.)

The pattern with age, with higher hazard ratios in children and young adults than in older adults, may be due to the study measuring time since the test rather than time since the infection, as older individuals are more likely to have been infected many years previously. But it may reflect true differences, as age at infection may influence the risk of progression to disease [3, 26]. The findings by age are similar to those observed in a large study in Denmark in the 1950s and 60s [27].

## Conclusion

We have shown that the risk of TB following remote Mtb infection persists well beyond 10 years, most likely driven by the remote infection. For those who are infected, remote infections can be an important source of TB disease even in a high incidence setting. By addressing this gap in evidence in TB epidemiology, crucial discussions on whether and how TB research and policy should prioritise the challenge of TB following remote Mtb infection should be better informed.

## Supporting information

S1 Appendix (DOCX)
