# Supplementary material for: Contribution of remote M.tuberculosis infection to tuberculosis disease: A 30-year population study
Source: PLoS One. 2023 Jan 27;18(1):e0278136. doi: 10.1371/journal.pone.0278136 (PMC9882759; doi:10.1371/journal.pone.0278136)
Supplement: S1 Appendix — (DOCX) [file pone.0278136.s001.docx]

**S1 Appendix for Contribution of remote *M.tuberculosis* infection to tuberculosis disease: a 30-year population study**

*Table 1: Individuals included in the different TST induration groups with characteristics and source of follow-up information*

|  |  | TST induration | | | | | |
| --- | --- | --- | --- | --- | --- | --- | --- |
|  |  | <5mm | | 18-20mm | | >20mm | |
|  |  | N | % | N | % | N | % |
|  |  |  |  |  |  |  |  |
| Identified |  | 52,633 | 100 | 3,005 | 100 | 1,629 | 100 |
|  |  |  |  |  |  |  |  |
| Age at TST |  |  |  |  |  |  |  |
|  | < 15 years | 35,443 | 67.3 | 449 | 14.9 | 316 | 19.4 |
|  | 15-29 years | 8,867 | 16.9 | 715 | 23.8 | 353 | 21.7 |
|  | 30-44 years | 4,132 | 7.9 | 733 | 24.4 | 364 | 22.3 |
|  | 45-59 years | 2,690 | 5.1 | 792 | 26.4 | 383 | 23.5 |
|  | ≥ 60 years | 1,501 | 2.9 | 316 | 10.5 | 213 | 13.1 |
|  |  |  |  |  |  |  |  |
| Sex | Female | 29,194 | 55.5 | 1,563 | 52.0 | 956 | 58.7 |
|  |  |  |  |  |  |  |  |
| Date of TST |  |  |  |  |  |  |  |
|  | 1980-84 (1^st^ survey) | 36,643 | 69.6 | 2,115 | 70.4 | 1,030 | 63.2 |
|  | 1985-89 (2^nd^ survey) | 15,990 | 30.4 | 890 | 29.6 | 599 | 36.8 |
|  |  |  |  |  |  |  |  |
|  |  |  |  |  |  |  |  |
| Area or residence (south to north) | |  |  |  |  |  |  |
|  | 1 (rural + truck-stop) | 6,999 | 13.3 | 280 | 9.3 | 197 | 12.1 |
|  | 2 (rural) | 10,434 | 19.8 | 483 | 16.1 | 297 | 18.2 |
|  | 3 (peri-urban) | 9,552 | 18.2 | 522 | 17.4 | 290 | 17.8 |
|  | 4 (urban) | 8,440 | 16.0 | 496 | 16.5 | 238 | 14.6 |
|  | 5 (rural + trading area) | 9,861 | 18.7 | 821 | 27.3 | 450 | 27.6 |
|  | 6 (rural + border) | 7,347 | 14.0 | 403 | 13.4 | 157 | 9.6 |
|  | |  |  |  |  |  |  |
| Follow-up information available | | 43,056 | 81.8 | 2,449 | 81.5 | 1,608 | 98.7 |
|  | |  |  |  |  |  |  |
| Last information (for those with follow-up): | |  |  |  |  |  |  |
|  | Whole population survey | 28,076 | 65.2 | 1,644 | 67.1 | 171 | 10.6 |
|  | Sample population surveys | 6,249 | 14.5 | 387 | 15.8 | 42 | 2.6 |
|  | Baseline census | 2,896 | 6.7 | 160 | 6.5 | 38 | 2.4 |
|  | Demographic surveillance | 4,079 | 9.5 | 123 | 5.0 | 88 | 5.5 |
|  | Follow-up of >20mm | 0 | 0 | 0 | 0 | 1,253 | 77.9 |
|  | Other | 1,756 | 4.1 | 135 | 5.5 | 16 | 1.0 |

*Table 2: Rate of tuberculosis by baseline induration size and time since the tuberculin skin test*

|  |  | TST <5mm | | |  | TST 18-20mm | | |  | TST >20mm | | |
| --- | --- | --- | --- | --- | --- | --- | --- | --- | --- | --- | --- | --- |
| Time since TST |  | TB | pyar | Rate/1000pyar  (95% CI) |  | TB | pyar | Rate/1000pyar  (95% CI) |  | TB | pyar | Rate/1000pyar  (95% CI) |
|  |  |  |  |  |  |  |  |  |  |  |  |  |
| < 2 years |  | 4 | 82,582 | 0.048 (0.018-0.13) |  | 10 | 4,697 | 2.13 (1.15-3.96) |  | 12 | 3,128 | 3.84 (2.18-6.75) |
| 2-9 years |  | 40 | 174,592 | 0.23 (0.17-0.31) |  | 14 | 9,630 | 1.45 (0.86-2.45) |  | 22 | 10,547 | 2.09 (1.37-3.17) |
| 10-19 years |  | 40 | 68,690 | 0.58 (0.43-0.79) |  | 5 | 2913 | 1.72 (0.71-4.12) |  | 13 | 9,489 | 1.37 (0.80-2.36) |
| ≥ 20 years |  | 43 | 40,769 | 1.05 (0.78-1.42) |  | 0 | 1,244 | 0.0 |  | 7 | 4,676 | 1.50 (0.71-3.14) |

pyar = person years at risk CI=confidence interval

*Table 3: Hazard ratios for tuberculosis by time since the tuberculin skin test by baseline induration size, compared to those with indurations of <5mm*

|  | TST 18-20mm | | TST >20mm | |
| --- | --- | --- | --- | --- |
| Time since TST | HR (95%CI) | | HR (95%CI) | |
|  | Crude | Adjusted | Crude | Adjusted |
|  |  |  |  |  |
| < 2 years | 45.95 (13.78-140.14) | 30.98 (8.65-110.90) | 79.19 (25.54-245.55) | 56.54 (16.45-194.25) |
| 2-9 years | 6.35 (3.45-11.66) | 3.55 (1.85-6.82) | 9.10 (5.41-15.32) | 4.91 (2.76-8.72) |
| 10-19 years | 2.95 (1.16-7.47) | 2.64 (0.99-7.05) | 2.35 (1.26-4.40) | 2.10 (1.00-4.42) |
| ≥ 20 years | - | - | 1.42 (0.64-3.16) | 2.66 (1.06-6.70) |

HR = hazard ratio, CI=confidence interval. Adjusted for age, sex, area

*Figure 1: Cumulative risk of tuberculosis by time since tuberculin skin test, and baseline TST induration size*

*Figure 2: Cumulative risk of tuberculosis by time since tuberculin skin test, induration size and HIV status at the time of tuberculosis diagnosis (a) HIV negative at the time of tuberculosis diagnosis (b) HIV positive at the time of tuberculosis diagnosis*

*Table 4: Hazard ratios for HIV positive and HIV negative tuberculosis by time since the tuberculin skin test comparing those with indurations >17mm to those with indurations of <5mm*

|  | HIV negative at time of TB | | | |  | HIV positive at time of TB | | | |
| --- | --- | --- | --- | --- | --- | --- | --- | --- | --- |
|  | TB | | HR (95%CI) | |  | TB | | HR (95%CI) | |
|  | <5mm | >17mm | Crude | Adjusted |  | <5mm | >17mm | Crude | Adjusted |
| Time since TST |  |  |  |  |  |  |  |  |  |
| < 2 years | 1 | 3 | 31.66 (3.29-304.34) | 34.35 (2.87-411.26) |  | 0 | 0 | - | - |
| 2-9 years | 22 | 14 | 5.51 (2.82-10.76) | 2.63 (1.28-5.44) |  | 3 | 1 | 2.88 (0.30-27.73) | 10.97 (1.14-105.42) |
| 10-19 years | 16 | 7 | 2.42 (1.00-5.89) | 1.69 (0.62-4.57) |  | 12 | 4 | 1.85 (0.60-5.72) | 2.38 (0.70-8.17) |
| ≥ 20 years | 8 | 2 | 1.72 (0.37-8.11) | 2.40 (0.43-13.28) |  | 23 | 3 | 0.90 (0.27-2.99) | 1.03 (0.29-3.69) |

HR = hazard ratio, CI=confidence interval

Adjusted for age at vaccination (<15, 15-29, 30+ years). Additional adjustment for sex and area made little difference to the results. Note that the low number of HIV positive tuberculosis in the first years after vaccination is due to lack of testing as well as low prevalence of HIV.

*Figure S3: Cumulative risk of tuberculosis by time since initial tuberculin skin test, induration size and age at the time of the skin test (a) 0-14 years of age (b) 15-29 years of age (c) ≥ 30 years of age*

*Table 5: Hazard ratios for tuberculosis by time since the tuberculin skin test comparing those with initial indurations >17mm to those with indurations of <5mm, by age at the time of the skin test*

|  | Age < 15 years | | | | Age 15-29 years | | | | | Age ≥ 30 years | | | | |  | |
| --- | --- | --- | --- | --- | --- | --- | --- | --- | --- | --- | --- | --- | --- | --- | --- | --- |
|  | <5mm | >17mm | HR (95%CI) | | <5mm | >17mm | HR (95%CI) | | <5mm | | >17mm | HR (95%CI) | |  | |  |
|  | TB | TB | Crude | Adjusted | TB | TB | Crude | Adjusted | TB | | TB | Crude | Adjusted | *p** | |  |
|  |  |  |  |  |  |  |  |  |  | |  |  |  |  | |  |
| Time since TST |  |  |  |  |  |  |  |  |  | |  |  |  |  | |  |
| < 2 years | 0 | 4 | - | - | 1 | 5 | 39.78 (4.65-340.53) | 46.99 (5.44-406.02) | 3 | | 13 | 12.23 (3.49-42.92) | 11.25 (3.17-39.90) | 0.015 | |  |
| 2-9 years | 13 | 4 | 10.12 (3.30-31.05) | 9.39 (3.01-29.33) | 11 | 9 | 5.21 (2.16-12.58) | 5.42 (2.21-13.26) | 16 | | 23 | 3.19 (1.69-6.04) | 3.03 (1.57-5.82) | 0.23 | |  |
| 10-19 years | 22 | 4 | 3.50 (1.21-10.15) | 3.17 (1.03-9.81) | 12 | 8 | 2.48 (1.02-6.07) | 2.55 (0.96-6.74) | 6 | | 6 | 1.20 (0.39-3.72) | 1.29 (0.38-4.44) | 0.40 | |  |
| ≥ 20 years | 30 | 2 | 1.44 (0.34-6.03) | 2.27 (0.51-10.21) | 10 | 4 | 1.87 (0.59-5.95) | 2.37 (0.70-8.05) | 3 | | 1 | 0.49 (0.051-4.71) | 0.46 (0.034-6.12) | 0.56 | |  |

HR = hazard ratio, CI=confidence interval, Adjusted for sex, area, **p* from likelihood ratio test for interaction (from unadjusted model)

*Table 6: Hazard ratios for tuberculosis by sex, by time since the tuberculin skin test comparing those with indurations >17mm to those with indurations of <5mm*

|  | Female | | | |  | Male | | | |  | *p* |
| --- | --- | --- | --- | --- | --- | --- | --- | --- | --- | --- | --- |
|  | TB | | HR (95%CI) | |  | TB | | HR (95%CI) | |  | interaction |
|  | <5mm | >17mm | Crude | Adjusted |  | <5mm | >17mm | Crude | Adjusted |  |  |
| Time since TST |  |  |  |  |  |  |  |  |  |  |  |
| < 2 years | 1 | 10 | 106.52 (13.64-832-10) | 142.80 (16.32-1249.67) |  | 3 | 12 | 41.72 (11.77-147-84) | 15.37 (3.86-61.12) |  | 0.38 |
| 2-9 years | 24 | 24 | 8.61 (4.89-15.17) | 6.07 (3.25-11.37) |  | 16 | 12 | 6.53 (3.09-13.80) | 2.40 (1.04-5.51) |  | 0.37 |
| 10-19 years | 20 | 13 | 3.44 (1.71-6.92) | 3.53 (1.59-7.84) |  | 20 | 5 | 1.48 (0.55-3.93) | 1.07 (0.36-3.16) |  | 0.14 |
| ≥ 20 years | 23 | 4 | 1.11 (0.38-3.20) | 1.18 (0.38-3.68) |  | 20 | 3 | 1.16 (0.35-3.92) | 1.74 (0.50-6.12) |  | 0.96 |

HR = hazard ratio, CI=confidence interval

Adjusted for age at vaccination (<15, 15-29, 30+ years). Additional adjustment for area made little difference to the results.

*Figure 4: Cumulative risk of tuberculosis by time since tuberculin skin test, induration size, and sex*

*Table 7: Rate of unique and linked tuberculosis by tuberculin skin test induration size*

|  |  | TST <5mm | | |  | TST >17mm | | |
| --- | --- | --- | --- | --- | --- | --- | --- | --- |
|  |  | TB | pyar | Rate/1000pyar  (95% CI) |  | TB | pyar | Rate/1000pyar  (95% CI) |
|  |  |  |  |  |  |  |  |  |
| Unique TB |  | 10 | 72,735 | 0.14 (0.074-0.26) |  | 6 | 11,560 | 0.52 (0.23-1.16) |
| Linked TB |  | 15 | 72,735 | 0.21 (0.12-0.34) |  | 3 | 11,560 | 0.26 (0.084-0.81) |

pyar = person years at risk CI=confidence interval

`

*Table S8: Hazard ratios for unique and linked tuberculosis after 1998 for those with indurations >17mm compared to those with indurations of <5mm*

|  | HR (95%CI) | | |
| --- | --- | --- | --- |
|  | Crude | Adjusted for age | Adjusted age, sex and area |
|  |  |  |  |
| Unique TB | 3.78 (1.37-10.38) | 5.27 (1.72-16.12) | 6.56 (1.96-22.99) |
| Linked TB | 1.25 (0.36-4.35) | 1.75 (0.46-6.65) | 2.26 (0.57-8.99) |
